# Can Tin Enhance the Electrocatalytic Activity of Fe–N_***x***_–C Materials toward the Oxygen Reduction Reaction?

**DOI:** 10.1021/acsaem.5c01351

**Published:** 2025-07-17

**Authors:** Nicolò Giulini, Mohsin Muhyuddin, Carmelo Lo Vecchio, Vincenzo Baglio, Luca Beverina, Carlo Santoro

**Affiliations:** † Department of Materials Science, 9305 University of Milano-Bicocca, U5 building, Via Cozzi 55, Milan 20125, Italy; ‡ 201828 Institute for Advanced Energy Technologies “Nicola Giordano” CNR-ITAE>, Via Salita S. Lucia sopra Contesse 5, Messina 98126, Italy

**Keywords:** atomically dispersed electrocatalysts, oxygen reduction reaction, Fe−N_*x*_−C, Fe−Sn bimetallic system, phthalocyanine

## Abstract

Hydrogen fuel cells are essential for addressing the energy transition process. However, the use of expensive platinum-based electrocatalysts poses a significant challenge for large-scale commercial deployment. Although Pt appears necessary to enhance the sluggish oxygen reduction reaction (ORR) kinetics, extensive research has been conducted aiming to replace it. Particularly, transition metals coordinated with nitrogen atoms and embedded in a conductive carbon framework (TM–N_*x*_–C) demonstrated promising results. In particular, Fe–N_*x*_–C electrocatalysts have shown superior electrocatalytic activity. Other monometallic or bimetallic systems have been less studied or have shown lower ORR metrics. Recently, a few reports have claimed the employment of tin (Sn–N_*x*_–C) as a beneficial secondary active site for fabricating electrocatalysts with good activity in both acidic and alkaline environments. In this scenario, the present work aimed to synthesize monometallic Sn–N_*x*_–C and Fe–N_*x*_–C, and bimetallic Fe–Sn–N_*x*_–C electrocatalysts following a simple, direct, and straightforward preparation method. Particularly, the corresponding tin- and iron-phthalocyanine precursors were blended with a conductive carbon substrate and subjected to a pyrolysis treatment at 600 or 800 °C. The obtained materials were thoroughly characterized and tested for ORR in both acidic and alkaline environments using a rotating ring disk electrode (RRDE). Sn-based electrocatalysts showed less electrocatalytic activity compared to Fe-based ones. Furthermore, a negligible or negative effect of tin co-functionalization was observed in each bimetallic sample. Although the proper blend of Sn and Fe precursors in the sample Sn/Fe(3:1)­Pc_600 led to an increased limiting current value compared to the iron counterpart, the other kinetic metrics were slightly negatively affected, especially in an acid electrolyte. Thus, the obtained results suggest that Sn co-functionalization seems to offer no noticeable enhancement in electrocatalytic activity.

## Graphical abstract

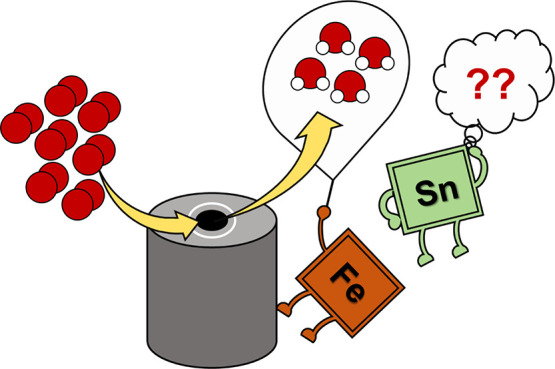

## 1 Introduction

Renewable energy (RE) resources play a central role in the energy transition.^1^ Hydrogen as an energy carrier has the potential to become a cost-effective solution for large-scale RE storage, transport, and export.^2^ Through the use of fuel cell (FC) technology, H_2_ can be efficiently oxidized into water molecules, transforming its chemical potential into electricity.^3^ Nevertheless, the use of expensive platinum-based electrocatalysts (ECs) poses a significant challenge for their large-scale commercial deployment.^4,5^ Nowadays, proton exchange membrane (PEM) FCs operating in acid electrolytes are the most developed technology.^6,7^ Pt within the ECs is necessary for considerably enhancing both the anodic and cathodic reactions. Particularly, the cathodic oxygen reduction reaction (ORR) requires high loading of the noble metal to overcome the sluggish kinetics and high overpotentials, substantially heightening the overall cost of FCs.^8^ Extensive research on enhancing the ECs’ ORR activity has been conducted, aiming to reduce the Pt content and, possibly, completely replace it.^9–14^

Recently, due to the development of anion exchange membranes (AEMs), FCs operating in alkaline environments are gaining great attention.^15–18^ Indeed, the alkaline electrolyte allows substituting Pt-based ORR ECs with platinum group metal-free (PGM-free) materials based on earth-abundant transition metals (TMs), including Fe, Co, Ni, Mn, Cu, etc.^19–22^ Atomically dispersed TMs coordinated with nitrogen in the form of TM–N_*x*_ (*x* = 2, 3, 4) embedded within a conductive carbon framework (TM–N_*x*_–C) have demonstrated exceptional activity for ORR in such environments.^23,24^ Other forms of TMs, such as metallic nanoparticles, oxides, or carbides, are less desirable as they tend to promote unwanted peroxides (H_2_O_2_), which are detrimental to the FC’s durability.^25^ Different synthetic strategies to achieve TM–N_*x*_–C-based electrocatalysts are presented in the literature, with the easiest, least sophisticated, least expensive, and easily scalable synthetic route consisting of integrating via pyrolysis aza-macrocyclic precursors containing the metal of interest into a carbon matrix.^26,27^ Particularly, involving metal-phthalocyanines (Pcs) as a source of single-metal nitrogen-rich molecules appeared to be beneficial.^28^ Moreover, pyrolysis is considered one of the most reliable methodologies to induce favorable and robust active sites in TM–N_*x*_–C with atomic-level homogeneity.^26,29^ The temperature of the heat treatment plays a pivotal role, as recently highlighted in the literature.^30^

Among TM–N_*x*_–C-based ECs, iron-containing materials are of interest, particularly in a porphyrin-like Fe–N_4_ coordination, which is considered one of the most selective and active configurations that lead to a direct 4e^–^ reduction pathway in ORR.^25,31^ Extensive studies on such materials have been reported and reviewed in the past decade.^32,33^ Due to the already complex nature of the monometallic system, bimetallic N-doped carbon has been less studied. However, the addition of a second metal center to Fe–N–C electrocatalysts could be beneficial in terms of active site formation, activity, or stability of the final material.^34,35^

The focus is currently centered on *d*-group metals such as Co or Mn.^36–39^ Nonetheless, other TMs may play a pivotal role, and this is the case of tin (Sn), which has recently captured much attention. Indeed, being a *p*-block element, tin is earth-abundant, environmentally compatible, low-cost, and highly available. Moreover, Sn shows high electrical conductivity, resistance to corrosion in sulfuric acid, good wettability, and distinct oxophilicity due to its high valence *p*-band. All these combined features make tin highly promising for electrocatalytic applications, especially in the form of Pt–Sn alloys.^40^ In a recent paper, Luo et al. showed the possible role of Sn in ORR, including its integration into a real fuel cell system.^41^ Other studies report the electrocatalytic activity of Sn–N_*x*_–C materials toward ORR, showing that they are more prone to forming hydrogen peroxide rather than reducing oxygen in a four-electron fashion.^42,43^ Only a few reports combine Fe and Sn at an atomically dispersed level into Fe–Sn–N_*x*_–C materials, taking advantage of their synergistic application, especially in alkaline environment experiments.^44,45^

This work aims to report the synthesis of new monometallic Sn–N_*x*_–C and Fe–N_*x*_–C, and bimetallic Fe–Sn–N_*x*_–C ECs for ORR both in acidic and alkaline environments, starting from the corresponding metal-phthalocyanine precursors blended with a conductive carbon substrate and involving pyrolysis as a reliable strategy to fabricate ECs. The chosen synthetic route is simple, direct, and straightforward and involves metal-containing aza macrocycles that are already in the TM-N_4_ desired form; moreover, the controlled pyrolytic treatment is useful to integrate both the Sn–N_*x*_ and Fe–N_*x*_ active sites into the highly graphitic carbon matrix. Additionally, this route has not yet been proposed to investigate Fe–Sn–N_*x*_–C electrocatalysts. A comprehensive chemical-physical characterization of the obtained materials has been provided. Finally, the electrocatalytic activity of the synthesized material through ORR in acidic and alkaline media has been evaluated via rotating ring-disk electrode experiments and compared to the monometallic counterparts.

## 2 Materials and Methods

### 2.1 Electrocatalysts Fabrication: General Procedure

All the ECs were synthesized by a simple and cost-effective procedure. 100 mg of tin­(II) phthalocyanine (TCI) or iron­(II) phthalocyanine (Acros Organics) or a proper mixture of both of them (Table 1) were manually blended by an agate mortar with 400 mg of Ketjenblack EC-600 JD (KJB, Nanografi), keeping an overall ratio of 20% precursor and 80% carbon support (1:4 ratio). The mixture was then transferred to a ZrO_2_ ceramic jar, equipped with 5 mm ZrO_2_ balls, and homogenized by 30 min ball milling (Retsch MM 200) at 10 Hz. Afterward, each sample was equally divided into two batches of the same weight and subjected to pyrolysis heat treatment. The mixtures were transferred onto an alumina boat and inserted into a quartz tube, equipped with an atmosphere-controlled flange system installed in a horizontal tube furnace (Nabertherm). One batch was pyrolyzed at 600 °C and the other one at 800 °C, both for 1 h (300 °C h^−1^ ramp rates for both heating and cooling) under a N_2_ atmosphere, after purging the tube with the inert gas for 30 min. All the samples were collected without any significant loss in weight and labeled with the general name: mixture composition as a function of the metal precursor content_Pyrolysis temperature expressed in Celsius degrees (e.g., Fe/Sn (3:1)­Pc_600 for the sample composed of 15 wt % of iron­(II) phthalocyanine and 5 wt % of tin­(II) phthalocyanine, pyrolyzed at 600 °C). A sketch of the synthetic procedure is reported in Figure 1.

**Table 1:** Name Assigned to the Synthesized ECs (Sample Name) Before the Heat Treatment, and Their Composition Expressed in Weight Percentage (wt %)

	Precursors mixture composition					
	iron(II) phthalocyanine		tin(II) phthalocyanine		KJB	
Sample name	wt %	mg	wt %	mg	wt %	mg
FePc	20	100	/	/	80	400
SnPc	/	/	20	100	80	400
Fe/Sn(1:1)*Pc*	10	50	10	50	80	400
Fe/Sn(3:1)*Pc*	15	75	5	25	80	400

**Figure 1:**
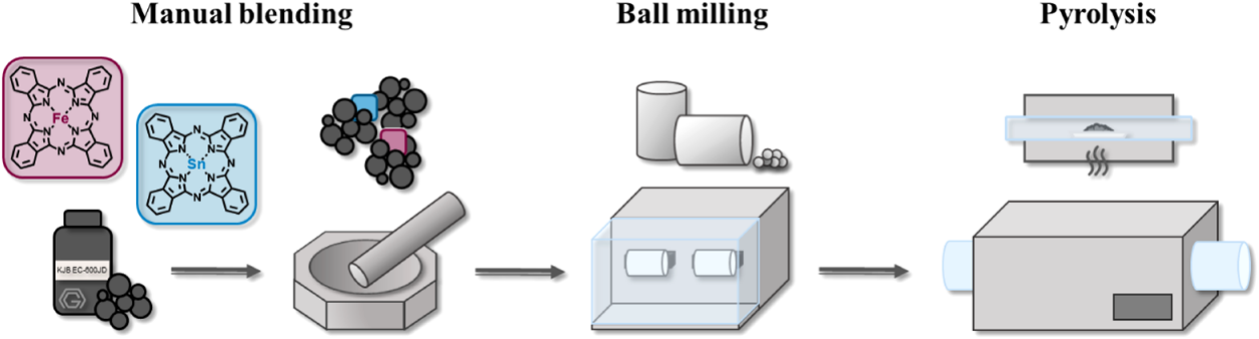
Sketch of the EC fabrication process. After proper manual blending of the right amount of iron­(II) and/or tin­(II) phthalocyanines, the mixture is subjected to homogenization through dry ball milling and, subsequently, through a pyrolysis heat treatment under an inert atmosphere.

### 2.2 Materials Characterization

The crystallographic features of the samples were investigated by X-ray diffraction (XRD, Rigaku Miniflex 600) performed in the 2θ range of 10–80°, with a step of 0.02° and a scanning rate of 3.0 °min^–1^. The structural integrity of the carbonaceous frameworks was studied through Raman spectroscopic measurements (LabRam, Jobin Yvon, France). A He–Ne laser (λ = 632.8 nm) was used to illuminate the samples via a microscope (BX40, Olympus, Japan) with an objective lens of 50× (numerical aperture of 0.60), while a silicon CCD (Sincerity, Jobin Yvon, France) was used for the signal collection at 200 K.

The surface properties of the ECs were examined using a Physical Electronics (PHI) 5800-01 X-ray photoelectron spectrometer, which utilized an Al Kα monochromatic X-ray source operating at a power of 350 W, as described in previous reports.^46,47^

Scanning transmission electron microscopy energy-dispersive X-ray spectroscopy (STEM-EDX) imaging was performed on a JEOL JEM 2100 Plus equipped with a four-element EDX detector, operating at 200 kV. Samples were prepared by drop casting an isopropanol-electrocatalyst dispersion (0.5 mg/mL) onto an ultrathin lacey carbon TEM grid. Images of three different areas were collected for each sample analyzed.

### 2.3 Electrochemical Measurements

The electrocatalytic activity of the synthesized samples for the ORR was investigated using a rotating ring-disk electrode (RRDE) setup, following established protocols.^48–50^ The experimental system featured a three-electrode configuration, consisting of a titanium wire as the counter electrode, a Ag-AgCl reference electrode (saturated KCl) for alkaline experiments, or a reversible hydrogen electrode (RHE) for acidic ones, and an RRDE (E6R2 series, Pine Instruments) as the working electrode. The working electrode was prepared by drop-casting the electrocatalyst ink onto the glassy carbon disk (0.2376 cm^2^) of the RRDE tip, with a mass loading of 0.6 mg cm^–2^, as reported in the literature.^45^ The ink was formulated by dispersing 5 mg of the electrocatalyst in a mixture of 985 μL of isopropanol (Alfa Aesar) and 15 μL of Nafion D-520 (5 wt %, Alfa Aesar), followed by 10 min of sonication (50% cycle, 50% amplitude) using a probe sonicator. ORR activity was evaluated under acidic and alkaline conditions with oxygen-saturated 0.5 M H_2_SO_4(aq)_ and 0.1 M KOH_(aq)_, respectively, as the electrolytes.

The study presents all the potential values referenced according to a reversible hydrogen electrode (RHE) using eq 1 as follows:

1 $$E_{RHE}=E_{Ag/AgCl}+E_{Ag/AgCl}^{0}+0.0591\cdot pH$$

where *E*_Ag/AgCl_ is the measured working potential versus the Ag/AgCl electrode, whereas $E_{Ag/AgCl}^{0}$ is the standard potential of the Ag/AgCl reference. pH value is 13 for a 0.1 M KOH aqueous solution. For tests in acidic media, the conversion is not required since RHE was directly used as the reference electrode.

Linear sweep voltammograms (LSVs) were recorded at a scan rate of 5 mV s^–1^ within a potential window of 1.2 to 0 V vs RHE, while maintaining the RRDE ring potential at 1.2 V vs RHE. The electrode was rotated at a constant speed of 1600 rpm. Before LSV measurements, the electrocatalyst was conditioned through multiple cyclic voltammetry scans until a stable response was achieved. The percentage of peroxide produced and the number of electrons transferred (*n*) during the ORR were determined using the disk current (*I*_disk_) and ring current (*I*_ring_), as described in eqs 2 and 3, respectively. The RRDE collection efficiency (*N*) was 38%.

2 $$Peroxide\left(\%\right)=\frac{200\cdot \frac{I_{ring}}{N}}{I_{disk}+\frac{I_{ring}}{N}}$$

3 $$n=\frac{4I_{disk}}{I_{disk}+\frac{I_{ring}}{N}}$$

## 3 Results and Discussion

### 3.1 Structural Characterization

XRD measurements were performed on all of the synthesized samples to evaluate the crystallographic features of the bulk materials (Figure 2a,b). As expected, two broad peaks at ∼25° and ∼44° are appreciable in all the analyzed patterns. These peaks refer to (002) and (100) lattices of graphitic carbon, respectively, as already observed in previous works, and can be rationally explained by the high carbon content (80 wt %, Table 1) of the catalysts.^48,51,52^ Furthermore, additional peaks are present in most of the samples. Concerning the ECs SnPc_600 and SnPc_800, both clearly show the presence of two tin phases, particularly referred to as metallic and tin­(II) oxide.^53^ Apparently, the 800 °C pyrolyzed sample exhibits a higher content of SnO compared to that of the 600 °C counterpart, suggesting that temperatures play a role in phase formation. Considering the catalysts FePc_600 and FePc_800, the formation of the magnetite Fe_3_O_4_ phase is documented at 800 °C, while it is avoided at 600 °C, as already observed and described in other works.^30,54^ Interestingly, slightly different behavior is observed for the mixed Fe/Sn bimetallic ECs. Both Fe/Sn(1:1)­Pc and Fe/Sn(3:1)­Pc pyrolyzed at 600 °C are free of crystallographic patterns (except for the carbonaceous signals), proposing that the atomic distribution of the metals is retained. Besides, Fe/Sn(3:1)­Pc_800 only shows the formation of a magnetite phase, while the aggregation of Sn is prevented; on the contrary, Fe/Sn(1:1)­Pc_800 exhibits both the presence of Fe_3_O_4_ along with a metallic Sn phase. Observing these results, it is possible to speculate that the presence of iron can prevent tin aggregation when the material is treated at 600 °C and when treated at 800 °C with a Fe/Sn atomic ratio of 3:1. The same tendency is appreciable in the STEM-EDX analysis discussed in Section 3.2.

**Figure 2:**
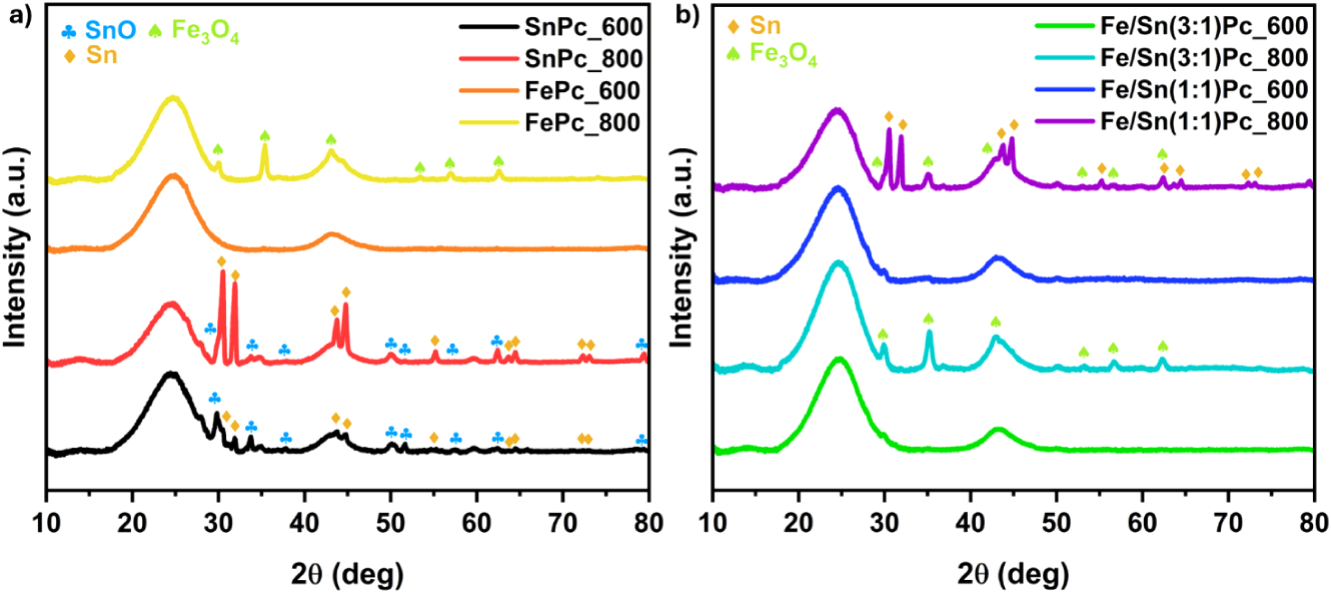
Diffraction patterns of the synthesized samples in the 10° to 80° 2θ range (a and b). The broad peaks at 25° and 44° refer to the (002) and (100) lattices of graphitic carbon. The peak identification of SnO, Sn, and Fe_3_O_4_ phases was performed according to the literature.^53,54^

Raman spectroscopy can be employed to further analyze the carbonaceous structure of the synthesized ECs. According to the literature, two characteristic absorption bands are commonly found in carbon-based materials: the G band (around 1580 cm^–1^) and the D band (around 1310 cm^–1^). Essentially, the G band relates to the degree of graphitization, while the D band reflects defects in the original lattice or the edges of graphene crystals. As is well established, the intensity ratio of the D to G bands (*I*_D_/*I*_G_) reflects the level of disorder in carbon-based materials.^55–57^ All synthesized samples exhibit an *I*_D_/*I*_G_ ratio greater than one, around 1.5, which suggests a high defect density introduced by the heat treatments, given that the ratio for pristine KJB is around 1.0 (Figure 3a,b). The latter feature enhances the ORR activity due to the altered electronic and chemical properties of the disrupted carbon structure.^58^

**Figure 3:**
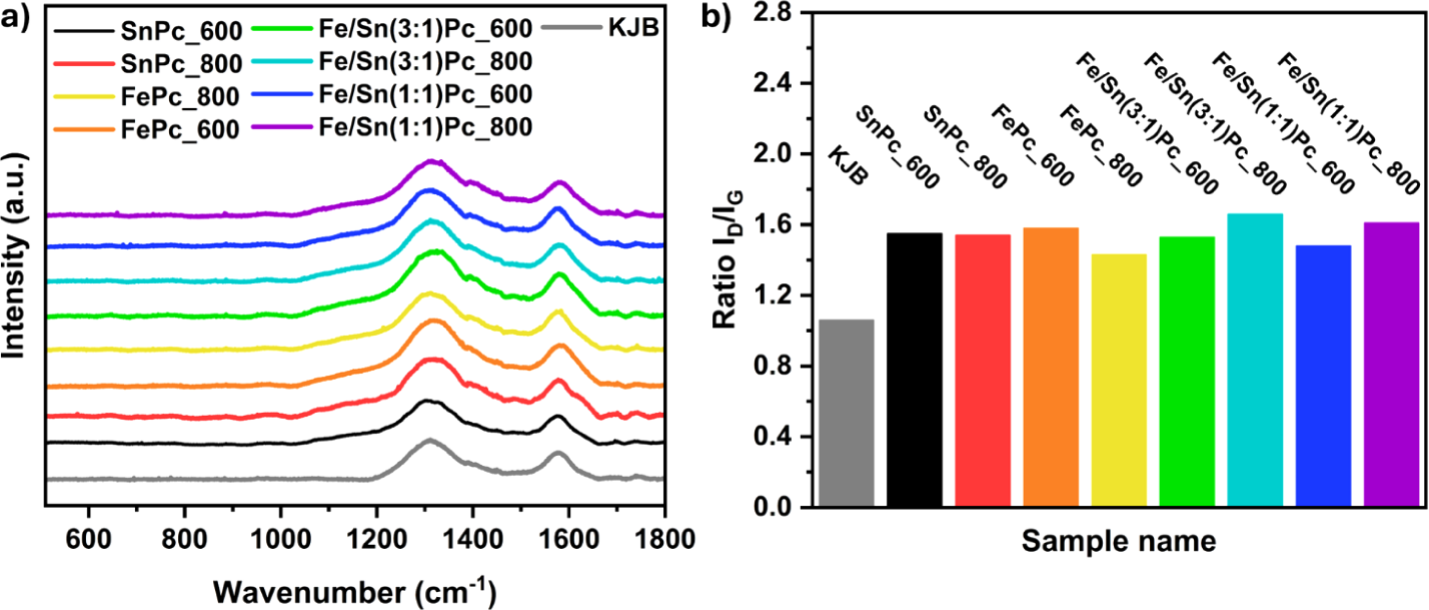
Raman spectroscopic features of the synthesized samples. (a) Collected Raman spectra. (b) Ratio between the normalized intensity of the D and G absorption bands (*I*_D_/*I*_G_).

### 3.2 Surface and Morphological Characterization

The surfaces of the Fe- and Sn-based ECs were analyzed using high-resolution N 1s and C 1s spectra. In the N 1s region, signals corresponding to five distinct nitrogen species can be identified: imine at 397.5 ± 0.1 eV, pyridinic-N at 398.4 ± 0.1 eV, N_*x*_–M (M = Fe, Sn) at 399.7 ± 0.1 eV, pyrrolic-N at 400.8 ± 0.1 eV, and graphitic-N at 402.3 ± 0.1 eV, as previously documented in the literature.^46,47,47–60^ The deconvolution spectra and a summary of nitrogen speciation are presented in Figure 4 and Table 2, respectively. Comparing the data from Figure 4, it is evident that the pyridinic/pyrrolic nitrogen ratio is significantly higher for the ECs treated at 600 °C (Figure 4a–d), whereas the intensity of the pyrrolic peak increases for those treated at 800 °C (Figure 4e–h). Another noteworthy observation, though less evident in the graph but highlighted in Table 2, is that the M–N_*x*_ interactions between the nitrogen species and metals (Fe, Sn) are more significant in the ECs treated at 600 °C than those treated at 800 °C at a maximum relative percentage of 41.0% for SnPc_600 followed by 31.2% for Fe/Sn(1:1)­Pc_600. Only a few imine and graphitic-N species were detected in most electrocatalysts, except for SnPc_600 and FePc_600.

**Figure 4:**
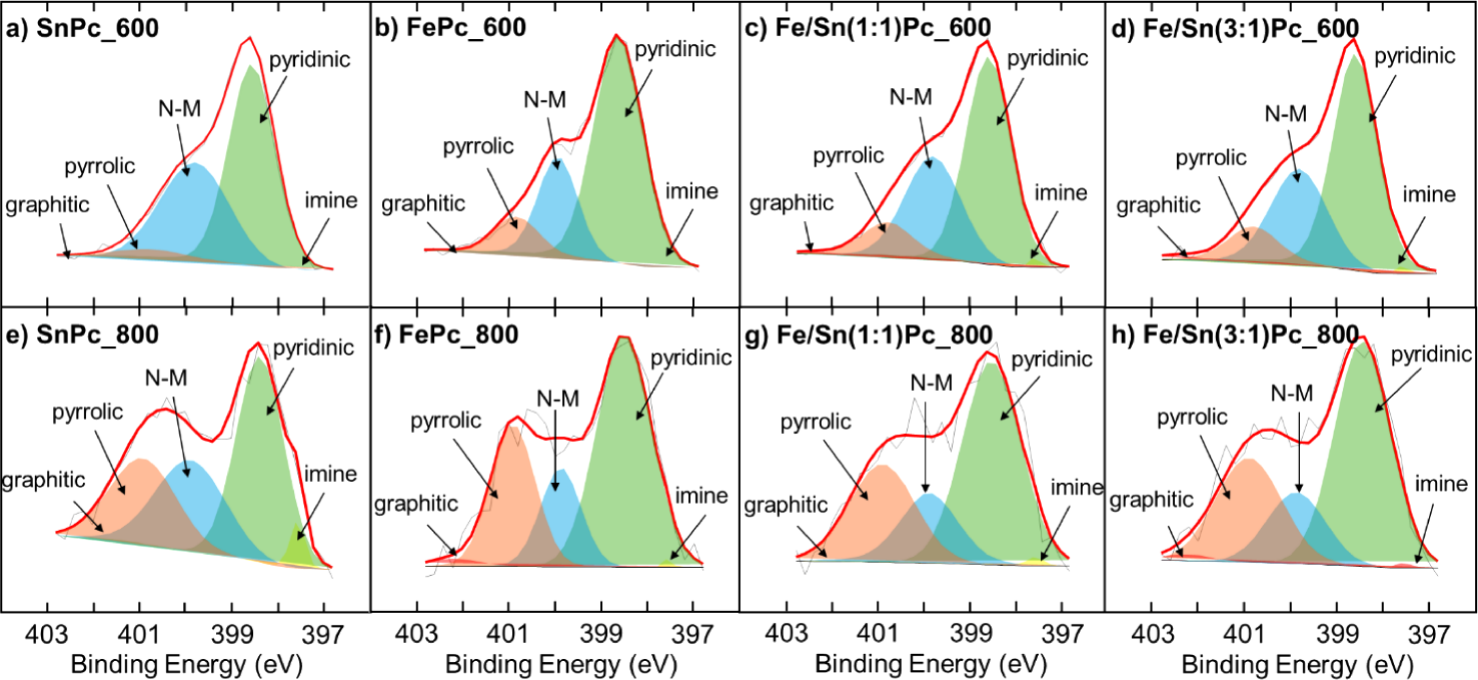
Comparison of XPS N 1s signals for the Fe and Sn-derived ECs treated at 600 °C (a–d) and 800 °C (e–h).

**Table 2:** Nitrogen Speciation from N 1s Deconvolution Spectra

Composition of *N* (relative %)						
Catalyst	*N* (at %)	Imine (397.5 ± 0.1 eV)	Pyridinic (398.4 ± 0.1 eV)	N_*x*_–M (M = Fe, Sn) (399.7 ± 0.1 eV)	Pyrrolic (400.8 ± 0.1 eV)	Graphitic (402.3 ± 0.1 eV)
SnPc_600	1.7	-	56.2	41.0	2.8	-
SnPc_800	1.3	3.2	43.7	28.0	24.9	0.2
FePc_600	1.7	-	66.9	23.6	9.5	-
FePc_800	0.8	0.3	54.1	17.1	27.0	1.5
Fe/Sn(1:1)Pc_600	1.7	6.9	51.6	31.2	9.9	0.4
Fe/Sn(1:1)Pc_800	0.7	0.7	52.4	19.1	27.8	-
Fe/Sn(3:1)Pc_600	1.7	5.7	54.7	28.5	10.2	0.9
Fe/Sn(3:1)Pc_800	0.8	0.3	52.6	17.1	28.7	1.3

These findings are valuable for directly correlating the N species with the ORR performance. Specifically, N-pyrrolic species cause a notable increase in H_2_O_2_ production during the ORR process, thus reducing the overall efficiency. On the other hand, pyridinic and graphitic nitrogen species generally improve ORR activity, driving the O_2_ reduction toward H_2_O production via a 4-electron mechanism.

The binding energies used for the C 1s deconvolution are as follows: 284.3 ± 0.1 eV for graphitic carbon, 285.0 ± 0.1 eV for secondary carbons such as carbon–nitrogen or carbon–oxide groups, 286.2 ± 0.1 eV for C–N_*x*_ defects, 287.1 ± 0.1 eV for alcohol and ether groups (C–OH/C–OC), 288.1 ± 0.1 eV for ketones or aldehydes (C=O), and 289.5 ± 0.1 eV for carboxylic species (COOH). The deconvolution spectra and a summary of carbon speciation are presented in Figure 5 and Table 3, respectively. Samples SnPc_600 (55.0%) and SnPc_800 (53.2%) exhibit the highest graphitic carbon content, which generally correlates to a lower ORR activity. No significant differences in composition were observed among the various electrocatalysts for other deconvoluted species, such as C–N defects and oxidized species.

**Figure 5:**
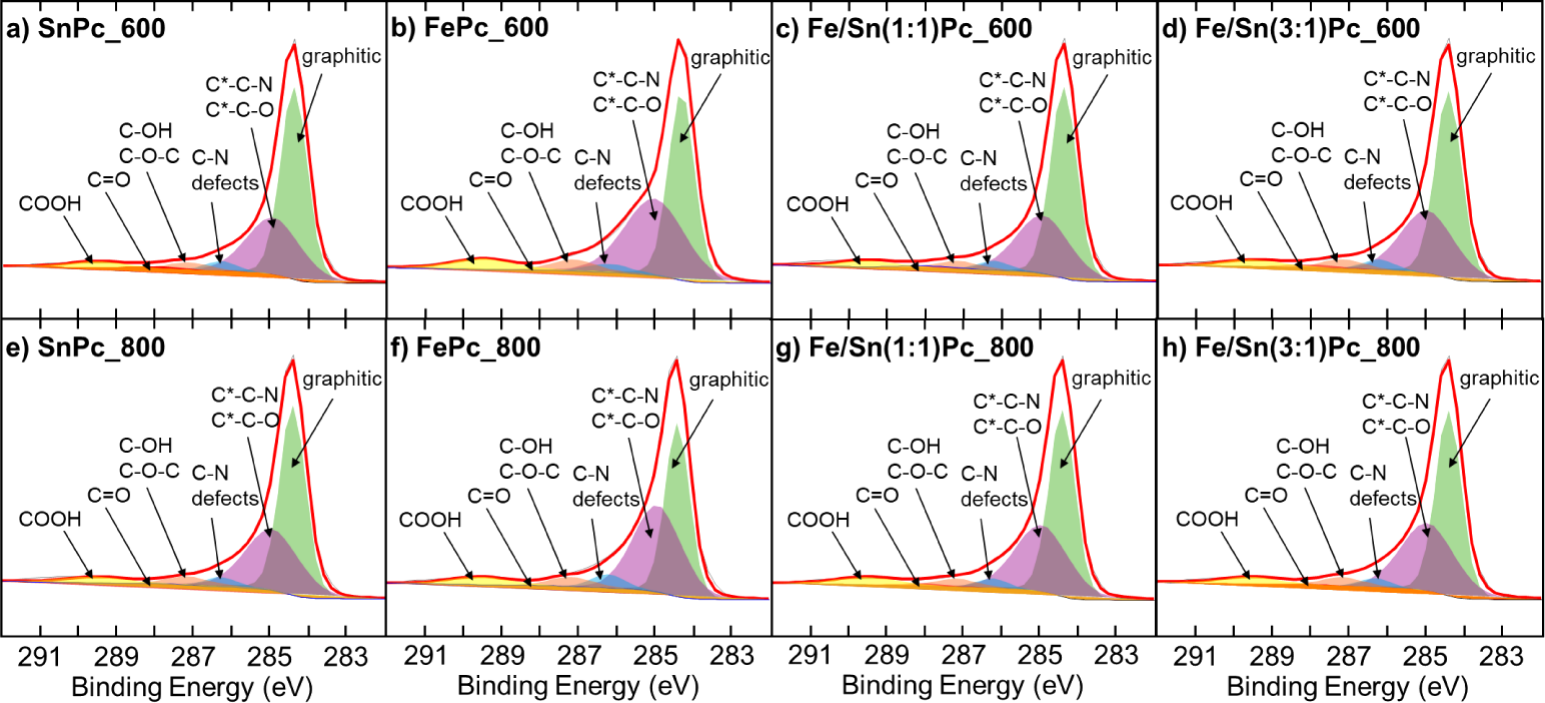
Comparison of XPS C 1s signals for the Fe- and Sn-derived ECs treated at 600 °C (a–d) and 800 °C (e–h).

**Table 3:** Carbon Speciation from C 1s Deconvolution Spectra

Composition of *C* (relative %)							
Catalyst	*C* (at %)	Graphitic (284.3 ± 0.1 eV)	Secondary carbons (285.0 ± 0.1 eV)	C–N_*x*_ Defects (286.2 ± 0.1 eV)	C–OH/C–OC (287.1 ± 0.1 eV)	C=O (288.1 ± 0.1 eV)	COOH (289.5 ± 0.1 eV)
SnPc_600	96.0	55.0	32.4	3.2	3.4	1.4	4.6
SnPc_800	97.5	53.2	35.1	3.0	3.9	0.7	4.1
FePc_600	96.0	44.1	41.3	3.1	4.7	0.3	6.5
FePc_800	97.5	43.0	42.5	4.8	4.3	0.4	5.0
Fe/Sn(1:1)Pc_600	95.9	53.8	33.2	3.1	3.8	1.5	4.6
Fe/Sn(1:1)Pc_800	96.5	50.4	36.6	3.2	3.7	1.3	4.8
Fe/Sn(3:1)Pc_600	95.7	51.1	35.0	3.3	4.3	1.7	4.5
Fe/Sn(3:1)Pc_800	96.7	49.4	38.0	2.9	4.0	0.7	4.9

STEM-EDX was performed on the samples to highlight the presence of nanoparticles (NPs) in various samples (Figures 6 and 7). As is known, transition metal-based NPs are usually not desired since they lead to an undesired 2e-transfer mechanism, especially in acid media.^25^ Regarding the tin-based materials, it is possible to appreciate the formation of both metallic and oxide nanoparticles, as confirmed by XRD analysis (Section 3.1), mostly rounded in shape and with diameters in the range 200–300 nm. Along with the NPs, dispersed Sn is present, with the SnPc_600 sample showing a higher content and better distribution of these active sites (Figure 6a,c). Alternatively, the iron-containing materials show the absence of NPs if the sample is treated at 600 °C, whereas Fe_3_O_4_ nanoparticles with a diameter of 50–100 nm are generated at 800 °C (Figure 6b,d).

**Figure 6:**
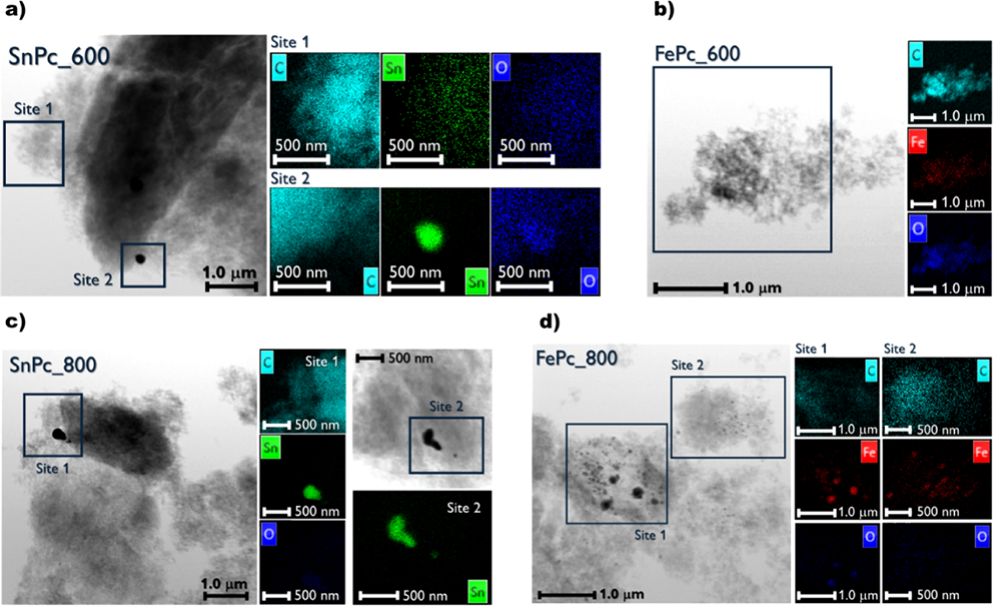
STEM (grayscale) and EDX mapping (black/colored survey) of the synthesized samples. (a) SnPc_600; (b) SnPc_800; (c) FePc_600; and (d) FePc_800. EDX survey legend: light blue dots are C signals, blue are O, green are Sn, and red are Fe.

**Figure 7:**
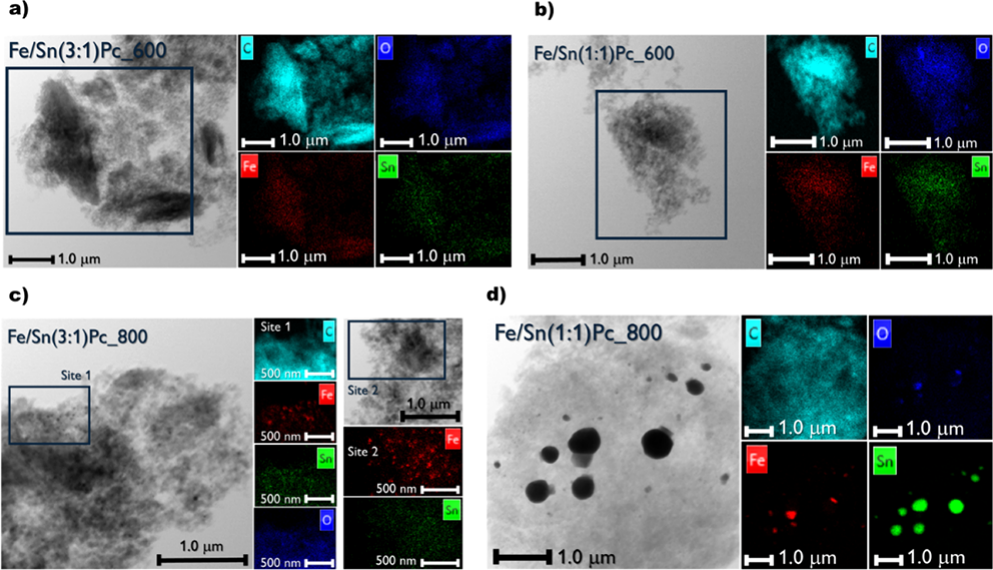
STEM (grayscale) and EDX mapping (black/colored survey) of the synthesized samples. (a) Fe/Sn(3:1)­Pc_600; (b) Fe/Sn(1:1)­Pc_600; (c) Fe/Sn(3:1)­Pc_800; and (d) Fe/Sn(1:1)­Pc_800. EDX survey legend: light blue dots are C signals, blue are O, green are Sn, and red are Fe.

Concerning the bimetallic systems, it is worth noticing that the presence of Fe appears to hinder or diminish the formation of Sn particles at 600 °C, at least up to the 1:1 ratio tested in the presented work (Figure 7a,b). Additionally, the same behavior is appreciable in the Fe/Sn (3:1)­Pc_800 electrocatalyst, where only the formation of iron oxide nanoparticles clearly occurs (Figure 7c). This evidence is confirmed by the absence of tin crystallographic signatures in the XRD plots reported in Figure 2. Interestingly, at 800 °C, this is no longer occurring when the relative weight percentage of tin increases up to a 1:1 Fe/Sn ratio. Indeed, the Fe/Sn (1:1)­Pc_800 material clearly shows the presence of considerable 400–600 nm rounded metallic tin NPs coupled with 100–300 nm polygonal magnetite particles, as can be appreciated in Figure 7d.

### 3.3 Electrocatalytic Activity Through RRDE Experiments

The synthesized materials discussed in this study were evaluated for ORR in both acid and alkaline media (O_2_-saturated 0.5 M H_2_SO_4_ or 0.1 M KOH, respectively; capacity correction applied) involving the RRDE experiment. The results are reported in Figure 8 and Table 4 (acid) and in Figure 9 and Table 5 (alkaline) and correspond to an EC loading of 0.6 mg cm^–2^. The choice of loading is aligned with what was reported by other groups, being aware that a thicker electrode may impact peroxide detection.^61–65^ Onset potential (*E*_on_) measured at 0.1 mA cm^–2^, half-wave potential (*E*_1/2_) calculated as the maximum of the first derivative of the linear sweep voltammetry curve, and limiting current density (*J*_lim_) registered at 0 V vs RHE are common electrocatalytic parameters acquired from RRDE. Moreover, the number of transferred electrons (*n*) and the percentage of peroxide produced are useful to evaluate the type of mechanism involved in O_2_ electroreduction (2-electrons, 4-electrons, or 2 × 2-electrons).^66^ Overall, 4-electron mechanisms are desirable. The obtained results are presented under these terms.

**Figure 8:**
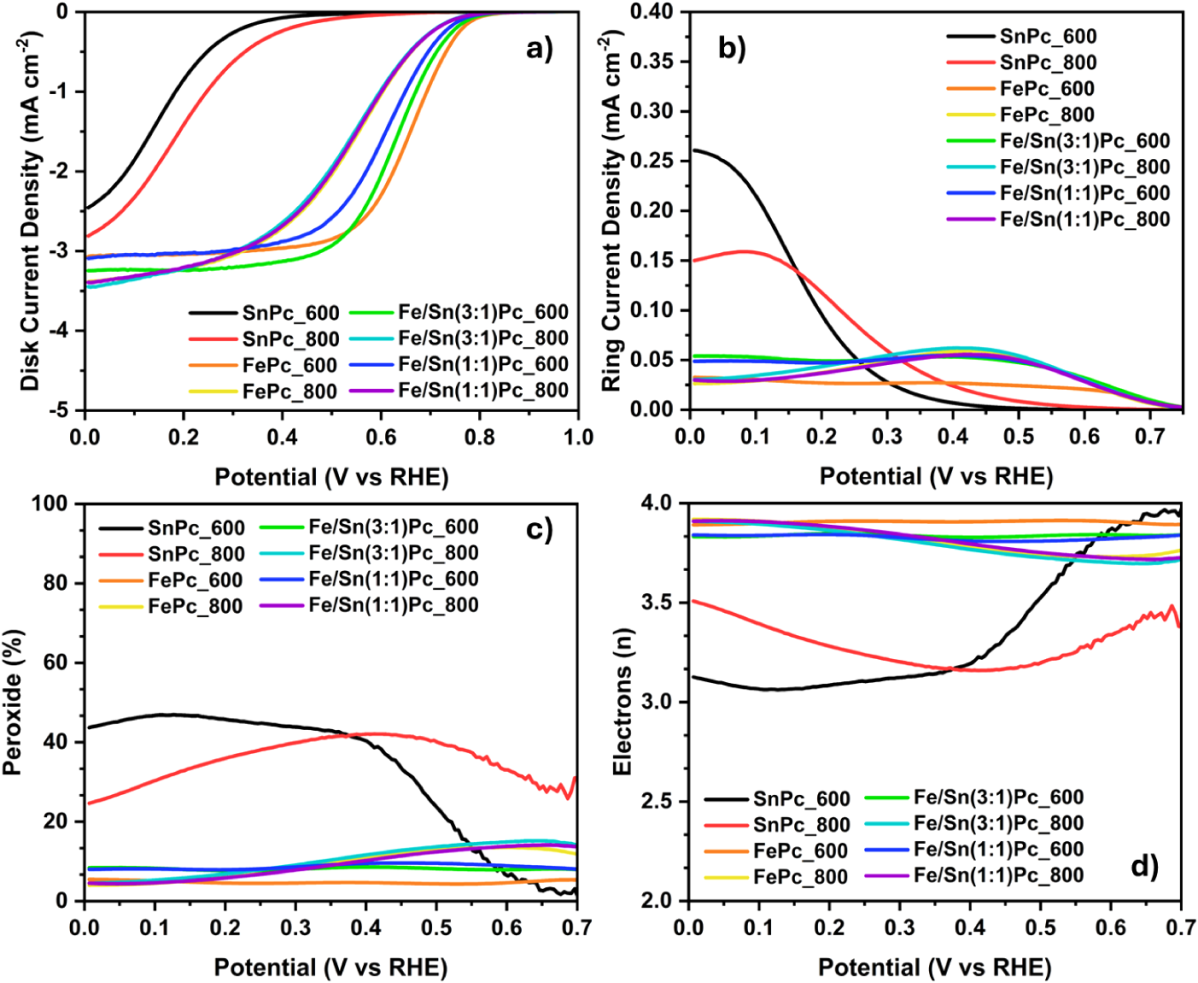
ORR electrocatalytic activity of the synthesized ECs in acid media (0.6 mg cm^–2^ loading in 0.5 M H_2_SO_4 (aq)_, oxygen saturated) recorded with an RRDE setup at 1600 rpm. (a) linear sweep voltammetry (LSV) at 5 mV s^–1^; (b) ring current densities; (c) trends of produced peroxide; (d) trends of the electrons transferred.

**Table 4:** Summary of ORR Activities in O_2_-Saturated 0.5 M H_2_SO_4 (Aq)_ Electrolytes

Sample Name	*E*_onset_ (V vs RHE)	*E*_1/2_ (V vs RHE)	*J*_limiting_ (mA cm^–2^)	% peroxide at 0 V vs RHE	no of e^–^ transf. at 0 V vs RHE
SnPc_600	0.372	0.146	/	44	3.1
SnPc_800	0.487	0.178	/	25	3.5
FePc_600	0.787	0.665	3.07	5	3.9
FePc_800	0.742	0.564	3.38	4	3.9
Fe/Sn(3:1)Pc_600	0.777	0.633	3.25	8	3.8
Fe/Sn(3:1)Pc_800	0.742	0.561	3.45	5	3.9
Fe/Sn(1:1)Pc_600	0.762	0.617	3.09	8	3.8
Fe/Sn(1:1)Pc_800	0.742	0.561	3.39	5	3.9

**Figure 9:**
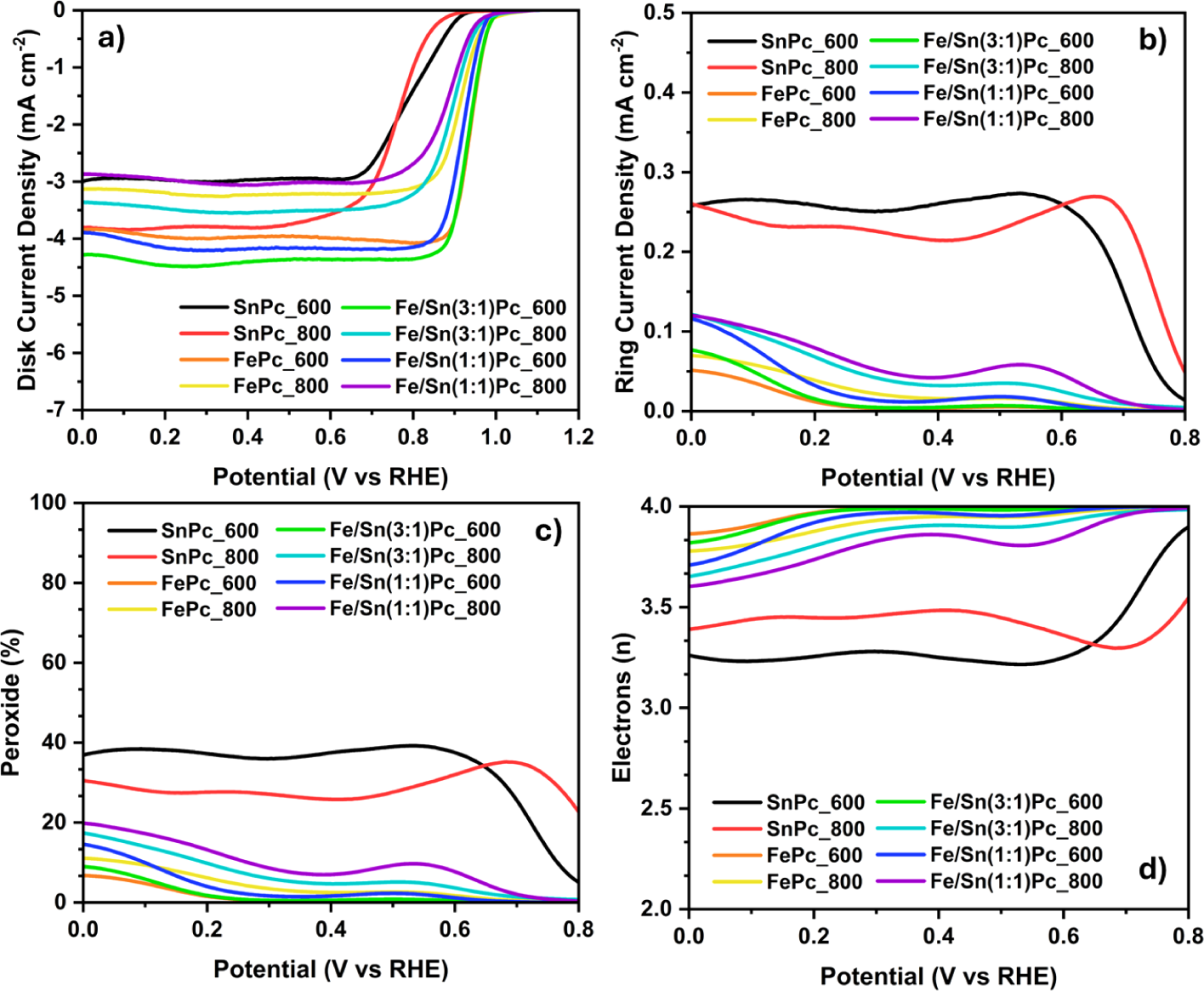
ORR electrocatalytic activity of the synthesized ECs in alkaline media (0.6 mg cm^–2^ loading in 0.1 M KOH_(aq)_, oxygen saturated) recorded with an RRDE setup at 1600 rpm. (a) Linear sweep voltammetry (LSV) at 5 mV s^–1^; (b) ring current densities; (c) trends of produced peroxide; (d) trends of the electrons transferred.

**Table 5:** Summary of ORR Activities in O_2_-Saturated 0.1 M KOH_(aq)_ Electrolyte

Sample Name	*E*_onset_ (V vs RHE)	*E*_1/2_ (V vs RHE)	*J*_limiting_ (mA cm^–2^)	% peroxide at 0 V vs RHE	no of e^–^ transf. at 0 V vs RHE
SnPc_600	0.913	0.744	3.00	37	3.3
SnPc_800	0.883	0.763	3.80	31	3.3
FePc_600	0.998	0.937	3.85	7	3.9
FePc_800	1.003	0.916	3.14	11	3.8
Fe/Sn(3:1)Pc_600	0.998	0.935	4.28	9	3.8
Fe/Sn(3:1)Pc_800	0.988	0.903	3.36	17	3.7
Fe/Sn(1:1)Pc_600	0.983	0.924	3.90	15	3.7
Fe/Sn(1:1)Pc_800	0.978	0.896	2.87	20	3.6

#### 3.3.1 ORR Activity in Acid Media

Concerning the acidic environment, the electrocatalytic activity of the materials synthesized in this work is listed in Table 4 and Figure 8. A classic sigmoidal shape is visible for all of the materials studied except for SnPc_600 and SnPc_800, which possess the highest overpotentials. Accordingly, the peroxide yield is high for both the ECs containing only SnPc as a metallic precursor, measuring between 20 and 40% (Figure 8c). For SnPc-derived ECs, the number of transferred electrons is around 3.2–3.5 on average, indicating the occurrence of both 4- and 2-electron mechanisms (Figure 8d). These results can be ascribed to the large excess of Sn nanoparticles that characterize the materials, as observed by STEM-EDX analysis (Section 3.2), but also to the low electrocatalytic activity of the active site of the type Sn–N_*x*_–C, as previously reported.^41^ In turn, iron-containing ECs have a much higher electrocatalytic activity toward ORR in acid media. Particularly, FePc_600 shows the best *E*_on_ and *E*_1/2_ of 0.787 and 0.665 V vs RHE, respectively, along with a *J*_lim_ of 3.07 mA cm^–2^. Further, an increase in the pyrolysis treatment to 800 °C applied to fabricate the FePc_800 sample leads to a decrease in ORR performance descriptors, along with an increase in peroxide production and of 2-electron reduction pathway contribution. Again, these results are aligned with the formation of metallic nanoparticles (Fe NPs) in the material investigated.

Regarding the bimetallic systems, only the materials pyrolyzed at 600 °C showed different electrocatalytic metrics compared to their iron monometallic counterparts. The sample Fe/Sn(3:1)­Pc_600 exhibits an *E*_on_ of 0.777 V vs RHE and an *E*_1/2_ of 0.633 V vs RHE; therefore, an appreciable decrease in the halfway potential occurs. Furthermore, the *J*_lim_ increases to 3.25 mA cm^–2^ (compared to 3.07 mA cm^–2^ of FePc_600), and this appears to be the sole beneficial effect since both the peroxide production and the number of electrons transferred are negatively affected by the co-functionalization. Increasing the Fe/Sn ratio to 1:1 in the Fe/Sn(1:1)­Pc_600 sample results in overall inferior ORR electrocatalytic activity, casting doubt on the potentially beneficial role of Sn. A further increase in the temperature of the heat treatment to 800 °C generates bimetallic ECs with nearly the same electrocatalytic behavior as the monometallic FePc_800 counterpart, suggesting that tin co-functionalization is unnecessary at that temperature.

#### 3.3.2 ORR Performance in Alkaline Media

Concerning the alkaline environment, a slightly different behavior is appreciable (Table 5 and Figure 9). The recorded performance is much higher in alkaline compared to acid media, and, generally, this occurs with PGM-free ECs.^67,68^ Indeed, the tin-containing electrocatalysts exhibit low performance compared to their iron counterparts, confirming the inevitability of Fe for carrying out ORR with superior kinetics, in agreement with recently reported literature.^41–45^ Interestingly, the SnPc_800 sample shows slightly higher *E*_1/2_ and *J*_lim_, lower peroxide production, and a higher number of electrons transferred on average compared to SnPc_600. However, both SnPc_600 and SnPc_800 have much lower activity and worse ORR descriptors compared to the other ECs investigated in alkaline media. As seen in the acid tests, FePc_600 is confirmed to be the best-performing material in terms of *E*_1/2_ (0.939 V vs RHE), peroxide produced, and the number of electrons transferred. Tin co-functionalization in the ratio Fe/Sn 3:1 (sample Fe/Sn(3:1)­Pc_600) slightly increases the *J*_lim_ (4.28 V vs RHE compared to 3.85 V vs RHE), although the peroxide produced is slightly higher and the electron transferred slightly lower. Other Fe/Sn ratios and/or a higher heat treatment temperature do not improve or even worsen the overall performance of FePc_600, as summarized in Table 5 and Figure 9.

## 4 Conclusion

The present work explored the possibility of involving tin as the primary active metal center in TM–N_*x*_–C-based ECs for ORR in both alkaline and acidic environments. Moreover, its ability to act as a co-catalyst in a bimetallic configuration in Fe–Sn–N_*x*_–C type materials was investigated, employing two different weight percentages of precursors (3:1 and 1:1 of Fe/Sn). Phthalocyanines were selected as organic precursors to functionalize a commercial conductive carbon substrate (KJB). Additionally, pyrolysis treatments at 600 and 800 °C were applied to embed the metal centers into the final materials. Electrocatalytic tests were conducted in the 0.5 M H_2_SO_4 (aq)_ electrolyte, demonstrating that tin alone does not appear to be a suitable element to fabricate high-performing ECs, probably due to the formation of considerable nanoparticles of metallic tin and tin oxide, as confirmed by XRD and STEM-EDX analysis. Mixing iron and tin in a ratio of 3:1 and pyrolyzing at 600 °C (Fe/Sn(3:1)­Pc_600 sample) proved to be beneficial in increasing the limiting current of the final material compared to the monometallic FePc_600 counterpart, but the other ORR descriptors were negatively affected (*E*_on_, *E*_1/2_, percentage of peroxide, and electrons transferred). The same behavior was recorded in 0.1 M KOH_(aq)_ tests over all of the synthesized materials, confirming the increased *J*_lim_ for Fe/Sn(3:1)­Pc_600, but lower values for most of the other metrics were obtained. As a result, Sn co-functionalization seems to offer no noticeable enhancement in the electrocatalytic activity of Fe–N_*x*_–C.

## References

[1] Gielen D., Boshell F., Saygin D., Bazilian M. D., Wagner N., Gorini R. (2019). The Role of Renewable Energy in the Global Energy Transformation. En. Strat. Rev..

[2] Dawood F., Anda M., Shafiullah G. M. (2020). Hydrogen
Production for Energy: An
Overview. Int. J. Hydrogen Energy.

[3] Staffell I., Scamman D., Velazquez Abad A., Balcombe P., Dodds P. E., Ekins P., Shah N., Ward K. R. (2019). The Role of Hydrogen
and Fuel Cells in the Global Energy System. Energy Environ. Sci..

[4] Sealy C. (2008). The Problem
with Platinum. Mater. Today.

[5] Pollet B. G., Kocha S. S., Staffell I. (2019). Current Status
of Automotive Fuel
Cells for Sustainable Transport. Curr. Opin.
Electrochem..

[6] Peighambardoust S. J., Rowshanzamir S., Amjadi M. (2010). Review of the Proton Exchange Membranes
for Fuel Cell Applications. Int. J. Hydrogen
Energy.

[7] Jiao K., Xuan J., Du Q., Bao Z., Xie B., Wang B., Zhao Y., Fan L., Wang H., Hou Z., Huo S., Brandon N. P., Yin Y., Guiver M. D. (2021). Designing
the next Generation of Proton-Exchange Membrane Fuel Cells. Nature.

[8] Ma Z., Cano Z. P., Yu A., Chen Z., Jiang G., Fu X., Yang L., Wu T., Bai Z., Lu J. (2020). Enhancing
Oxygen Reduction Activity of Pt-Based Electrocatalysts: From Theoretical
Mechanisms to Practical Methods. Angew. Chem.,
Int. Ed..

[9] Ren X., Lv Q., Liu L., Liu B., Wang Y., Liu A., Wu G. (2020). Current Progress of Pt and Pt-Based Electrocatalysts Used for Fuel
Cells. Sustainable Energy Fuels.

[10] Zhang J., Yuan Y., Gao L., Zeng G., Li M., Huang H. (2021). Stabilizing Pt-Based
Electrocatalysts for Oxygen Reduction Reaction:
Fundamental Understanding and Design Strategies. Adv. Mater..

[11] Tong M., Wang L., Fu H. (2021). Designed Synthesis and Catalytic
Mechanisms of Non-Precious Metal Single-Atom Catalysts for Oxygen
Reduction Reaction. Small Methods.

[12] Huang L., Zaman S., Tian X., Wang Z., Fang W., Xia B. Y. (2021). Advanced Platinum-Based
Oxygen Reduction Electrocatalysts
for Fuel Cells. Acc. Chem. Res..

[13] Liu M., Xiao X., Li Q., Luo L., Ding M., Zhang B., Li Y., Zou J., Jiang B. (2022). Recent Progress
of Electrocatalysts for Oxygen Reduction in Fuel Cells. J. Colloid Interface Sci..

[14] Li X., Wang D., Zha S., Chu Y., Pan L., Wu M., Liu C., Wang W., Mitsuzaki N., Chen Z. (2024). Active Sites Identification and Engineering
of M-N-C Electrocatalysts
toward Oxygen Reduction Reaction. Int. J. Hydrogen
Energy.

[15] Merle G., Wessling M., Nijmeijer K. (2011). Anion Exchange Membranes for Alkaline
Fuel Cells: A Review. J. Membr. Sci..

[16] Varcoe J. R., Atanassov P., Dekel D. R., Herring A. M., Hickner M. A., Kohl P. A., Kucernak A. R., Mustain W. E., Nijmeijer K., Scott K., Xu T., Zhuang L. (2014). Anion-Exchange Membranes
in Electrochemical Energy Systems. Energy Environ.
Sci..

[17] Gottesfeld S., Dekel D. R., Page M., Bae C., Yan Y., Zelenay P., Kim Y. S. (2018). Anion Exchange Membrane Fuel Cells:
Current Status and Remaining Challenges. J.
Power Sources.

[18] Dekel D. R. (2018). Review
of Cell Performance in Anion Exchange Membrane Fuel Cells. J. Power Sources.

[19] Huang X., Shen T., Zhang T., Qiu H., Gu X., Ali Z., Hou Y. (2020). Efficient Oxygen Reduction
Catalysts of Porous Carbon
Nanostructures Decorated with Transition Metal Species. Adv. Energy Mater..

[20] Hossen M., Hasan S., Sardar R. I., Haider J. B., Tammeveski M. K., Atanassov P. (2023). State-of-the-Art and Developmental Trends in Platinum
Group Metal-Free Cathode Catalyst for Anion Exchange Membrane Fuel
Cell (AEMFC). Appl. Catal., B.

[21] Wu G., Santandreu A., Kellogg W., Gupta S., Ogoke O., Zhang H., Wang H.-L., Dai L. (2016). Carbon Nanocomposite
Catalysts for Oxygen Reduction and Evolution Reactions: From Nitrogen
Doping to Transition-Metal Addition. Nano Energy.

[22] Wu G., Zelenay P. (2013). Nanostructured Nonprecious
Metal Catalysts for Oxygen
Reduction Reaction. Acc. Chem. Res..

[23] Osmieri L. (2019). Transition
Metal–Nitrogen–Carbon (M–N–C) Catalysts
for Oxygen Reduction Reaction. Insights on Synthesis and Performance
in Polymer Electrolyte Fuel Cells. ChemEngineering.

[24] Sarapuu A., Lilloja J., Akula S., Zagal J. H., Specchia S., Tammeveski K. (2023). Recent Advances
in Non-Precious Metal Single-Atom Electrocatalysts
for Oxygen Reduction Reaction in Low-Temperature Polymer-Electrolyte
Fuel Cells. ChemCatChem.

[25] Artyushkova K., Serov A., Rojas-Carbonell S., Atanassov P. (2015). Chemistry
of Multitudinous Active Sites for Oxygen Reduction Reaction in Transition
Metal–Nitrogen–Carbon Electrocatalysts. J. Phys. Chem. C.

[26] Ouyang C., Wang X. (2020). Recent Progress in
Pyrolyzed Carbon Materials as Electrocatalysts
for the Oxygen Reduction Reaction. Inorg. Chem.
Front.

[27] Luo E., Chu Y., Liu J., Shi Z., Zhu S., Gong L., Ge J., Choi C. H., Liu C., Xing W. (2021). Pyrolyzed M–Nx
Catalysts for Oxygen Reduction Reaction: Progress and Prospects. Energy Environ. Sci..

[28] Yang S., Yu Y., Gao X., Zhang Z., Wang F. (2021). Recent Advances in
Electrocatalysis with Phthalocyanines. Chem.
Soc. Rev..

[29] Chen Z., Higgins D., Yu A., Zhang L., Zhang J. (2011). A Review on
Non-Precious Metal Electrocatalysts for PEM Fuel Cells. Energy Environ. Sci..

[30] Muhyuddin M., Berretti E., Mirshokraee S. A., Orsilli J., Lorenzi R., Capozzoli L., D’Acapito F., Murphy E., Guo S., Atanassov P., Lavacchi A., Santoro C. (2024). Formation of the Active
Site Structures during Pyrolysis Transformation of Fe-Phthalocyanine
into Fe-Nx-C Electrocatalysts for the Oxygen Reduction Reaction. Appl. Catal., B.

[31] Facchin A., Zerbetto M., Gennaro A., Vittadini A., Forrer D., Durante C. (2021). Oxygen Reduction Reaction at Single-Site
Catalysts: A Combined Electrochemical Scanning Tunnelling Microscopy
and DFT Investigation on Iron Octaethylporphyrin Chloride on HOPG. ChemElectroChem.

[32] Shen H., Thomas T., Rasaki S. A., Saad A., Hu C., Wang J., Yang M. (2019). Oxygen Reduction
Reactions of Fe-N-C
Catalysts: Current Status and the Way Forward. Electrochem. Energy Rev..

[33] Wang W., Jia Q., Mukerjee S., Chen S. (2019). Recent Insights into the Oxygen-Reduction
Electrocatalysis of Fe/N/C Materials. ACS Catal..

[34] Sahraie N. R., Kramm U. I., Steinberg J., Zhang Y., Thomas A., Reier T., Paraknowitsch J.-P., Strasser P. (2015). Quantifying the Density
and Utilization of Active Sites in Non-Precious Metal Oxygen Electroreduction
Catalysts. Nat. Commun..

[35] Luo F., Wagner S., Onishi I., Selve S., Li S., Ju W., Wang H., Steinberg J., Thomas A., Kramm U. I., Strasser P. (2021). Surface Site
Density and Utilization of Platinum Group
Metal (PGM)-Free Fe–NC and FeNi–NC Electrocatalysts
for the Oxygen Reduction Reaction. Chem. Sci..

[36] Samad S., Loh K. S., Wong W. Y., Sudarsono W., Lee T. K., Wan Daud W. R. (2020). Effect of Various
Fe/Co Ratios and
Annealing Temperatures on a Fe/Co Catalyst Supported with Nitrogen-Doped
Reduced Graphene Oxide towards the Oxygen Reduction Reaction. J. Alloys Compd..

[37] Yuan S., Cui L.-L., Dou Z., Ge X., He X., Zhang W., Asefa T. (2020). Nonprecious Bimetallic Sites Coordinated
on N-Doped Carbons with Efficient and Durable Catalytic Activity for
Oxygen Reduction. Small.

[38] Wang J., Liu C., Li S., Li Y., Zhang Q., Peng Q., Tse J. S., Wu Z. (2022). Advanced Electrocatalysts
with Dual-Metal
Doped Carbon Materials: Achievements and Challenges. Chem. Eng. J..

[39] Zhang Y.-L., Goh K., Zhao L., Sui X.-L., Gong X.-F., Cai J.-J., Zhou Q.-Y., Zhang H.-D., Li L., Kong F.-R., Gu D.-M., Wang Z.-B. (2020). Advanced Non-Noble Materials in Bifunctional
Catalysts for ORR and OER toward Aqueous Metal–Air Batteries. Nanoscale.

[40] Gao M., Zhang X., Dai S., Wang K.-W. (2024). Tin as a Co-Catalyst
for Electrocatalytic Oxidation and Reduction Reactions. Inorg. Chem. Front.

[41] Luo F., Roy A., Silvioli L., Cullen D. A., Zitolo A., Sougrati M. T., Oguz I. C., Mineva T., Teschner D., Wagner S., Wen J., Dionigi F., Kramm U. I., Rossmeisl J., Jaouen F., Strasser P. (2020). P-Block Single-Metal-Site Tin/Nitrogen-Doped
Carbon Fuel Cell Cathode Catalyst for Oxygen Reduction Reaction. Nat. Mater..

[42] Sapkota P., Lim S., Aguey-Zinsou K.-F. (2023). Tin-Nitrogen/Carbon
for Superior
Oxygen Reduction Reaction at Fuel Cell Cathode. Int. J. Hydrogen Energy.

[43] Jiang R., Zhi Q., Jin Y., Liu W., Chen B., Yang B., Li W., Qi D., Wang K., Sun T., Jiang J. (2024). Atomic Sn
Sites Supported on N-Doped Porous Carbon for Accelerating the Oxygen
Reduction Reaction. Catal. Sci. Technol..

[44] Negro E., Bach Delpeuch A., Vezzù K., Nawn G., Bertasi F., Ansaldo A., Pellegrini V., Dembinska B., Zoladek S., Miecznikowski K., Rutkowska I. A., Skunik-Nuckowska M., Kulesza P. J., Bonaccorso F., Di Noto V. (2018). Toward Pt-Free Anion-Exchange Membrane Fuel Cells:
Fe–Sn Carbon Nitride–Graphene Core–Shell Electrocatalysts
for the Oxygen Reduction Reaction. Chem. Mater..

[45] Mazzucato M., Gavioli L., Balzano V., Berretti E., Rizzi G. A., Badocco D., Pastore P., Zitolo A., Durante C. (2022). Synergistic
Effect of Sn and Fe in Fe–Nx Site Formation and Activity in
Fe–N–C Catalyst for ORR. ACS Appl.
Mater. Interfaces.

[46] Lo
Vecchio C., Aricò A. S., Baglio V. (2018). Application of Low-Cost
Me-N-C (Me = Fe or Co) Electrocatalysts Derived from EDTA in Direct
Methanol Fuel Cells (DMFCs). Materials.

[47] Lo
Vecchio C., Aricò A. S., Monforte G., Baglio V. (2018). EDTA-Derived
CoNC and FeNC Electro-Catalysts for the Oxygen Reduction Reaction
in Acid Environment. Renewable Energy.

[48] Muhyuddin M., Testa D., Lorenzi R., Vanacore G. M., Poli F., Soavi F., Specchia S., Giurlani W., Innocenti M., Rosi L., Santoro C. (2022). Iron-Based
Electrocatalysts Derived
from Scrap Tires for Oxygen Reduction Reaction: Evolution of Synthesis-Structure-Performance
Relationship in Acidic, Neutral and Alkaline Media. Electrochim. Acta.

[49] Zago S., Bartoli M., Muhyuddin M., Vanacore G. M., Jagdale P., Tagliaferro A., Santoro C., Specchia S. (2022). Engineered Biochar
Derived from Pyrolyzed Waste Tea as a Carbon Support for Fe-N-C Electrocatalysts
for the Oxygen Reduction Reaction. Electrochim.
Acta.

[50] Giulini N., Muhyuddin M., Mattiello S., Sassi M., Lo Vecchio C., Baglio V., Berretti E., Lavacchi A., Fazio E., Beverina L., Santoro C. (2024). Repurposing Discarded Porphyrin Waste
as Electrocatalysts for the Oxygen Reduction Reaction. Electrochim. Acta.

[51] Sevilla M., Fuertes A. B. (2006). Catalytic Graphitization of Templated Mesoporous Carbons. Carbon.

[52] Inagaki, M. , New Carbons - Control of Structure and Functions; Elsevier, 2000.

[53] He F., Xu Q., Zheng B., Zhang J., Wu Z., Zhong Y., Chen Y., Xiang W., Zhong B., Guo X. (2020). Synthesis
of Hierarchical Sn/SnO Nanosheets Assembled by Carbon-Coated Hollow
Nanospheres as Anode Materials for Lithium/Sodium Ion Batteries. RSC Adv..

[54] Peng H., Mo Z., Liao S., Liang H., Yang L., Luo F., Song H., Zhong Y., Zhang B. (2013). High Performance Fe-
and N- Doped Carbon Catalyst with Graphene Structure for Oxygen Reduction. Sci. Rep..

[55] Ferrari A. C., Robertson J. (2000). Interpretation of Raman Spectra of Disordered and Amorphous
Carbon. Phys. Rev. B.

[56] Cançado L. G., Jorio A., Ferreira E. H. M., Stavale F., Achete C. A., Capaz R. B., Moutinho M. V. O., Lombardo A., Kulmala T. S., Ferrari A. C. (2011). Quantifying Defects
in Graphene via Raman Spectroscopy
at Different Excitation Energies. Nano Lett..

[57] Malard L.
M., Pimenta M. A., Dresselhaus G., Dresselhaus M. S. (2009). Raman Spectroscopy
in Graphene. Phys. Rep..

[58] Zhu C., Shi Q., Xu B. Z., Fu S., Wan G., Yang C., Yao S., Song J., Zhou H., Du D., Beckman S. P., Su D., Lin Y. (2018). Hierarchically Porous M–N–C (M = Co and
Fe) Single-Atom Electrocatalysts with Robust MNx Active Moieties Enable
Enhanced ORR Performance. Adv. Energy Mater..

[59] Mercado R., Wahl C., En Lu J., Zhang T., Lu B., Zhang P., Lu J. Q., Allen A., Zhang J. Z., Chen S. (2020). Nitrogen-Doped Porous
Carbon Cages for Electrocatalytic Reduction
of Oxygen: Enhanced Performance with Iron and Cobalt Dual Metal Centers. ChemCatChem.

[60] Gokhale R., Chen Y., Serov A., Artyushkova K., Atanassov P. (2016). Direct Synthesis of Platinum Group
Metal-Free Fe-N-C
Catalyst for Oxygen Reduction Reaction in Alkaline Media. Electrochem. Commun..

[61] Bonakdarpour A., Lefevre M., Yang R., Jaouen F., Dahn T., Dodelet J.-P., Dahn J. R. (2008). Impact of Loading
in RRDE Experiments
on Fe–N–C Catalysts: Two- or Four-Electron Oxygen Reduction?. Electrochem. Solid-State Lett..

[62] Jaouen F., Dodelet J.-P. (2009). O2 Reduction Mechanism
on Non-Noble Metal Catalysts
for PEM Fuel Cells. Part I: Experimental Rates of O2 Electroreduction,
H2O2 Electroreduction, and H2O2 Disproportionation. J. Phys. Chem. C.

[63] Serov A., Tylus U., Artyushkova K., Mukerjee S., Atanassov P. (2014). Mechanistic
Studies of Oxygen Reduction on Fe-PEI Derived Non-PGM Electrocatalysts. Appl. Catal., B.

[64] Muhyuddin M., Friedman A., Poli F., Petri E., Honig H., Basile F., Fasolini A., Lorenzi R., Berretti E., Bellini M., Lavacchi A., Elbaz L., Santoro C., Soavi F. (2023). Lignin-Derived Bimetallic Platinum
Group Metal-Free Oxygen Reduction
Reaction Electrocatalysts for Acid and Alkaline Fuel Cells. J. Power Sources.

[65] Mazzucato M., Durante C. (2022). Insights on Oxygen Reduction Reaction to H2O2: The
Role of Functional Groups and Textural Properties on the Activity
and Selectivity of Doped Carbon Electrocatalysts. Curr. Opin. Electrochem..

[66] Ficca V. C. A., Santoro C., Placidi E., Arciprete F., Serov A., Atanassov P., Mecheri B. (2023). Exchange Current Density
as an Effective Descriptor of Poisoning of Active Sites in Platinum
Group Metal-Free Electrocatalysts for Oxygen Reduction Reaction. ACS Catal..

[67] Muhyuddin M., Mostoni S., Honig H. C., Mirizzi L., Elbaz L., Scotti R., D’Arienzo M., Santoro C. (2024). Enhancing Electrocatalysis:
Engineering the Fe–Nx–C Electrocatalyst for Oxygen Reduction
Reaction Using Fe-Functionalized Silica Hard Templates. ACS Appl. Energy Mater..

[68] Honig H. C., Mostoni S., Presman Y., Snitkoff-Sol R. Z., Valagussa P., D’Arienzo M., Scotti R., Santoro C., Muhyuddin M., Elbaz L. (2024). Morphological and Structural Design
through Hard-Templating of PGM-Free Electrocatalysts for AEMFC Applications. Nanoscale.

